# When Physics Meets Biology: Low and High-Velocity Penetration, Blunt Impact, and Blast Injuries to the Brain

**DOI:** 10.3389/fneur.2015.00089

**Published:** 2015-05-07

**Authors:** Leanne Young, Gregory T. Rule, Robert T. Bocchieri, Timothy J. Walilko, Jennie M. Burns, Geoffrey Ling

**Affiliations:** ^1^Security Engineering and Applied Sciences Sector, Applied Research Associates, Inc., Dallas, TX, USA; ^2^Center for Brain Health, University of Texas at Dallas, Dallas, TX, USA; ^3^Security Engineering and Applied Sciences Sector, Applied Research Associates, Inc., San Antonio, TX, USA; ^4^Silicon Valley Office, Applied Research Associates, Inc., Los Altos, CA, USA; ^5^Rocky Mountain Division, Applied Research Associates, Inc., Littleton, CO, USA; ^6^Department of Neurology, Uniformed Services University of the Health Sciences, Bethesda, MD, USA

**Keywords:** ballistics, blast, blunt trauma, traumatic brain injury

## Abstract

The incidence of traumatic brain injuries (TBI) in the US has reached epidemic proportions with well over 2 million new cases reported each year. TBI can occur in both civilians and warfighters, with head injuries occurring in both combat and non-combat situations from a variety of threats, including ballistic penetration, acceleration, blunt impact, and blast. Most generally, TBI is a condition in which physical loads exceed the capacity of brain tissues to absorb without injury. More specifically, TBI results when sufficient external force is applied to the head and is subsequently converted into stresses that must be absorbed or redirected by protective equipment. If the stresses are not sufficiently absorbed or redirected, they will lead to damage of extracranial soft tissue and the skull. Complex interactions and kinematics of the head, neck and jaw cause strains within the brain tissue, resulting in structural, anatomical damage that is characteristic of the inciting insult. This mechanical trauma then initiates a neuro-chemical cascade that leads to the functional consequences of TBI, such as cognitive impairment. To fully understand the mechanisms by which TBI occurs, it is critically important to understand the effects of the loading environments created by these threats. In the following, a review is made of the pertinent complex loading conditions and how these loads cause injury. Also discussed are injury thresholds and gaps in knowledge, both of which are needed to design improved protective systems.

## Introduction

A significant challenge facing researchers seeking to prevent, mitigate, diagnose or treat traumatic brain injuries (TBI) is the heterogeneity of the disease. In fact, the term “TBI” refers to a wide range of injuries caused by a variety of injury mechanisms and leading to a range of clinical consequences. Penetrating head injuries are typically associated with projectiles moving at high velocities, such as rifle bullets, or low velocities, such as knives. Closed-head injuries are typically associated with blunt force, such as being struck by a blunt instrument, overpressure from an explosion, or head acceleration. Because there is an intimate relationship between the mechanism of injury and the subsequent cascade of injury, research into appropriate prevention, diagnosis and treatment of TBI is a multidisciplinary endeavor.

The importance of researching TBI cannot be overstated. According to the Centers for Disease Control (CDC) approximately 1.7 million TBIs occur each year in the US with 52,000 fatalities and 275,000 hospitalizations (1). Investigators acknowledge that under-reporting is common so that the actual figure may be up to 10-fold higher. TBI accounts for 30% of injury-related deaths in the US (1). For the US military, statistics vary widely due to the difficulties associated with diagnosing mild TBI; however, reports indicate that 15 and 20% of returning veterans from Afghanistan and Iraq have head injuries (2). While most TBI victims recover fully within a few days to weeks, many continue to experience chronic symptoms of cognitive, emotional, and physical impairment for months or years (3).

Determining the mechanisms underlying the various types of TBI begins with studying the physics of the insult. TBI is a consequence of a physical insult that exceeds the capacity of the biological organism to tolerate. It is this physical insult that initiates the biochemical and pathological consequences that is brain injury.

This paper reviews the elements of physics of the different types of TBI. These elements are loading mechanisms associated with ballistic, blast, and blunt trauma and accelerative forces. For each, the physics of the interaction between the insult and head are described. Although injury criteria and injury prevention are largely considered to be engineering issues, rather than medical ones, it is the opinion of the authors that naiveté on the part of medical personnel regarding the state of the science in injury prevention does as great a disservice to TBI research as does naiveté on the part of engineers to the state of the science in TBI diagnosis and treatment. Thus, this paper includes a summary of the state of the science with respect to injury criteria and prevention derived from the present state of the understanding of TBI physics.

## Ballistics

Penetrating head wounds are typically categorized as resulting from low or high-velocity projectiles. The definitions of “low” and “high” vary. From a ballistics protection perspective, “low-velocity” is sometimes defined as <120 m/s, but British researchers define “low velocity” as below 335 m/s, and American researchers have also drawn the line anywhere between 610 m/s and 914 m/s (4). From a biomechanics perspective, the definition of “low” and “high” velocity is complicated by the close relationships among the velocity, mass and projected area of the penetrator. It is obvious that low-velocity penetrators would include knives or glass fragments. It is, perhaps, less intuitive that some pellets from, for example, a .38 caliber air rifle, may also result in a “low-velocity” penetration to the brain. A.38 caliber air rifle pellet weighing 8.25 grains must impact at a velocity of at least 101 m/s to perforate skin, leaving relatively little residual kinetic energy with which to penetrate the skull and damage the brain. By the time it does penetrate the skull, if it does, the damage it produces to brain matter will be relatively localized. By comparison, a round nose.38 caliber lead bullet weighing 113 grains can perforate skin at 58 m/s, leaving much more residual energy with which to penetrate the skull and underlying brain matter (5). Given the interdependency of velocity, mass, projectile construction and projected area, the terms “low velocity” and “high velocity” are, at the least, misleading. However, since the terms continue to be ubiquitous in both biomechanics and medical communities, perhaps the best way of defining “low velocity” and “high velocity” is by the nature of the injury, with “low velocity” penetrations characterized by highly localized tissue damage along the object’s trajectory, while “high velocity” penetrations generate both permanent and temporary cavities, resulting in damage beyond the immediate contact region between projectile and tissue (6).

### Low-velocity penetrations

Low-velocity penetrating wounds cause lacerations to the scalp, depressed skull fractures and localized brain tissue damage along the object’s travel path. The head is most commonly violated by low-velocity projectiles at weak points of the skull, where the bone is thinnest. These skull locations are the orbital roof, temporal squama and cribriform plate (nasal cavity). Other skull locations are still vulnerable but are more difficult to breach due to skull thickness (7, 8). In low-velocity wounds, the injury is made primarily by either crushing or cutting tissue along the path. Low-velocity projectiles with sharp edges, such as knives and glass, penetrate via a cutting mechanism whereas low-velocity projectiles without a sharp edge penetrate by way of kinetic energy deposition, crushing along the way (6). In either case, the wound tract dimensions are typically the dimensions of the penetrating object. Thus, the neurological deficits are ascribable to the brain regions directly affected along the foreign body’s penetration path. As only a relatively small portion of the brain region is affected, neurological deficits may be limited. However, if a particularly critical brain region, such as the midbrain, is involved, then even a very small foreign body may result in irreversible coma or death.

With knife wounds and low-velocity projectiles, such as air rifle pellets, where most of the energy of the projectile is expended prior to penetrating the skull, there may only be minimal local brain damage. If the projectile or skull fragment remains inside the cranial cavity and near the inner skull table, these patients may be candidates for surgical debridement. If there is a depressed skull fracture without foreign body penetration, then bone fragment spallation into the brain may still occur. If this happens, the patient usually remains conscious with minimal neurological deficit, but is at risk of an infection, epidural, subdural or subarachnoid hematoma (9). In the event of a subarachnoid hematoma, clots and debris may form in the basal cisterns, or fibrosis and adhesion of the meninges may occur, resulting in a disruption of cerebralspinal fluid flow in the subarachnoid space and leading to ventricular enlargement from obstructive hydrocephalus (10). Again, neurosurgical intervention is usually required for optimal clinical outcome. In general, survival and functional outcomes are better following low-velocity injuries than high.

### Low-velocity penetration thresholds

Injury thresholds from low-velocity penetration are not well characterized. For blunt impactors, the force required to penetrate the skull is dependent on the impactor mass, its acceleration and the thickness of the skull at the point of impact. For a given mass and acceleration (i.e., force), the primary determining factors are the projected area of the penetrator and the location of impact. The projected area affects the area over which the force of the penetrator is applied. If this is held constant, but the mass is changed, as with the previously discussed. 38 caliber pellets and lead bullets, the threshold for penetration changes. The energy required to penetrate the skull is a product of the applied force and the distance over which that force is applied. Thus, less energy is required to penetrate thinner parts of the skull. With respect to the location of impact, the thickest parts of the human skull are the posterior parietal and occipital skull followed by the temporal and frontal (11). Studies have shown that at the parietal bone, an impactor of 200–297 mm^2^ will penetrate with quasi-static forces ranging from 980 to 1334 N (12).

For sharp impactors, such as shards or knives, that penetrate via a cutting mechanism, soft tissue penetration is a function of mass, impact velocity, the presented area of the projectile and tissue density (13). Existing models for these types of impactors have not been validated for bone penetration. In fact, the threshold for skull penetration with thin sharp objects such as a knife or glass is not known. Similarly, the thresholds are unknown for most rounded projectiles, such as the nose of a low-velocity handgun bullet.

### High-velocity penetrations

Because they are both more common and more injurious, considerably more research has been dedicated to understanding the biomechanics and clinical implications of high-velocity penetrators. High-velocity penetration is characterized by high kinetic energy, and accompanying shock waves resulting in three distinct areas of tissue damage: (1) the wound tract, where tissue is lacerated and crushed, (2) an adjacent area of damaged tissues caused by shearing and stretching, and (3) a surrounding area with a lack of filling of small blood vessels and extravasation of blood (14). As with low-velocity wounding, the high-velocity projectile tract is a result of crushing and tearing tissue by the foreign body as it traverses brain tissue. The result is a permanent cavity. The diameter of this permanent cavity is a function of the penetrator’s dimensions and velocity. In contrast to low-velocity penetrators, the permanent cavity from a high-velocity deforming penetrator is typically a few times greater than the projectile diameter. This is due to the contribution of cavitation, which does not accompany low-velocity injury. In addition, the penetrator’s tumbling and yaw can contribute to the permanent cavity size. For a non-deforming, tumbling penetrator, the cavity is roughly the length of the penetrator. The permanent cavity from a fragmenting penetrator can be even larger, as it depends on the dispersal pattern of the fragments (15). The adjacent area of damaged tissues is called the temporary cavity. It is caused by large scale cavitation, i.e., a “transient displacement of tissue” (16) within brain tissue. It is a brief, compressive force that expands tangentially from the wound tract (4, 17). The creation and collapse of the temporary cavity leads to temporary dilation that stretches and shears brain parenchyma (18). Although transient, the pathology from the temporary cavity can be significant, much more so than directly from the projectile path (14).

There is a temptation to assume that the severity of injury from a ballistic penetration will be a function of kinetic energy alone. However, the larger of two projectiles of equivalent kinetic energy will typically crush more tissue (i.e., create a larger permanent cavity), while the smaller (faster) will stretch more tissue (i.e., create a larger temporary cavity). While there is a positive correlation between wound severity and both mass and velocity, factors such as the orientation of the projectile, fragmentation and deformation, projectile mass and the tissue tract itself also influence the size of both the permanent and temporary cavities (16).

In addition to the direct tissue effects characterized by the permanent and temporary cavities, there is an important additional risk of physiological consequences. For example, a large temporary cavity formed following a high-velocity bullet injury will lead to wide spread cerebral edema. This edema results in increased intracranial pressure (ICP) (18). With high ICP, brain tissue herniates. A particularly devastating event is downward herniation of the cerebrum into the posterior skull fossa leading to compression and then functional failure of the brainstem respiratory and cardiac centers, resulting in death (19).

### High-velocity penetration thresholds

Identifying a single threshold of high-velocity penetration injury is challenging because the projectile’s ability to cause trauma is dependent on the velocity and mass (energy), and shape and diameter (impact surface area) of the projectile. Most researchers use gelatin as the body tissue simulant for injury/wound quantification (20). Testing is typically accomplished by firing a projectile into a rectangular shaped simulant. This approach is an inappropriate TBI model as the brain is encased in an incompressible structure (skull) and is neither rectangular nor of uniform density. However, some investigators are now taking these issues into consideration. For example, Yoganandan and colleagues place a brain model made of silicone dielectric gel (Sylgard 527) and ballistic gelatin into a sphere that approximates the skull (20). The study uses two different projectiles, 9-mm and 25-caliber. Since the Sylgard gel is contained in a sphere and has material properties closer to the human brain, it is more representative of the human condition. Although this experimental approach still fails to replicate the heterogeneity of brain tissue, the pressure distributions and other results can be used with caution for determining brain injury thresholds. While no single brain injury threshold from ballistic projectiles exists, it is widely assumed that any skull and brain injury from a high-velocity bullet round will result in severe TBI leading to either profound long-term disabilities or death (21). For this reason, all ballistic head protection is designed to a zero penetration criterion, leaving behind-armor blunt trauma as the major risk of injury from ballistic threats that are defeated by helmets.

### Ballistic protection

Ballistic threats are commonly associated with war but can also occur in civilian assault cases. There is a long history of head protection systems developed for U.S. military personnel. Up until recently, the most common military helmets were made entirely out of DuPont’s Kevlar^®^, and weighed as much as 1.6 kg. The current generation of U.S. military helmets, such as the Advanced Combat Helmet is also constructed of a thermoset resin shell bonded to Kevlar^®^. The Enhanced Combat Helmet (ECH), in contrast, is constructed of ultra-high molecular weight polyethylene reinforced by carbon fibers (22). These new helmets are up to 15% lighter in weight than previous generations of helmets and achieve equivalent ballistic protection as the previous heavier models (23). The use of ultra high molecular weight polyethylene (UHMWPE) in the construction of the helmet shell continues to be explored to increase the level of ballistic protection beyond that of the ECH, without an increase in weight (22, 24). For law enforcement, a variety of head protection options are available, including various military helmets constructed from Kevlar^®^. Helmets are tested for ballistic protection using the National Institute of Justice (NIJ) 0106.01 protocol, which defines levels of protection as a function of weapon caliber and velocity to verify that no penetration occurs for either frontal or lateral impacts (25).

**Figure 1:**
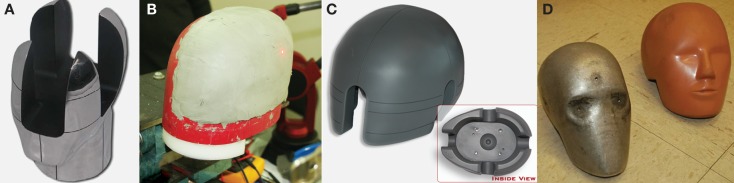
**Head forms used in ballistic and blunt trauma testing**. **(A)** NIJ ballistic head form for testing behind armor blunt trauma, constructed of aluminum, with a cavity filled with modeling clay. **(B)** PEEPsite ballistic head form for testing behind armor blunt trauma, designed out of concern for artifacts created by the vertical petals of the NIJ head form. **(C)** ISO head form for measuring blunt trauma, used in conjunction with the maximum translational peak acceleration criterion. (www.cadexinc.com) **(D)** Hybrid III head form for measuring blunt trauma used with the Head Injury Criterion. (Bass CR, Walilko TJ, Kent R. Evaluation of head surrogates for the assessment of explosive and ballistic injuries. Final report submitted to the Combatting Terrorism Technology Support Office by University of Virginia. April 2011. Contract Number W91CRB-09-0015).

If no penetration occurs, United States Department of Defense (DoD) protocols require that the helmets also be evaluated for prevention of behind-armor blunt trauma (26). This trauma results from back face deformation of the helmet when a foreign body strikes it. Although the projectile does not perforate the helmet, the material deformation can impact the head leading to blunt head trauma. To evaluate for behind-armor blunt trauma, the NIJ clay head form is used. It is constructed of aluminum, with a cavity that is filled prior to testing with Roma Plastilina No. 1 oil-based modeling clay (Figure 1A). This particular clay is plastic, so that it maintains its shape after impact, allowing for measurement of indentation depth (25). An indentation of 25 mm or less is accepted for front/back impacts, and an indentation of 16 mm or less is the acceptance criteria for lateral impacts (26). There are a number of shortcomings with respect to the NIJ head form. To begin, it is difficult to accurately measure the back face deformation. Next, the vertical petals of the head form create a rigid boundary condition that influences the back face deformation measurement. It is difficult to maintain the clay within the calibration specification, and the recipe for the clay has changed with time, so that the clay is stiffer for more recent batches (27). Perhaps most concerning of all, there remains no valid injury data that links clinical outcome to degree of back face deformation in clay.

As an alternative to the NIJ head form, the US Army Research Laboratory developed the PEEPsite head form (Figure 1B). This head form is based upon the same concept of measuring indentation into plastic clay. However, the head form is designed such that the rigid boundary condition imposed by the vertical petals in the NIJ head form is removed (27). Unfortunately, although the PEEPsite head form addresses the issue of rigid boundary conditions, issues with respect to clay calibration and achieving accurate measurements of back face deformation remain, as well as the lack of clinical validation.

## Blunt Trauma

Blunt trauma and acceleration cause injury by way of skull deformation, rotational motion and ICP (28). Both elastic and plastic localized skull deformations transmit localized stress to underlying tissues, resulting in strains to the neural or neurovascular tissues and leading to contusions at the site of impact. The subsequent rebound of the skull can separate the dura mater and skull, resulting in epidural hematomas (28). Because the brain is not rigidly attached to the interior of the skull, upon impact the motion of the brain lags behind that of the head and skull. As a result, another source of injuries is an impact between the inner table of the skull and the brain. When the head motion comes to a stop and moves back to its original orientation, the brain will then impact the skull on the opposite side of the original impact point (12, 29). Contusions at the point of impact are referred to as *coup* lesions, and contusions distal to the point of impact are referred to as *contrecoup* lesions. Both can be clinically significant.

Although rotational motions are known to be highly injurious, the mechanism of injury remains unclear. There are two dominant theories regarding how rotational motion injures the brain. One theory proposes that tissue shearing occurs at the interfaces between adjacent tissues moving at different rates as a result of different densities (30). In support of this theory, research has shown that diffuse injuries caused by rotation are greatest near the surface of the brain, where the rotational motion is the greatest; they are reduced near the center of mass, where the rotational motion is the least (28, 30). Thus, low levels of rotational inertial loading primarily result in cortical injuries, whereas more significant rotational motions can cause injuries deep into brain regions within the diencephalon.

A second theory on rotational motion is that the injuries are caused by the relative inability of the brain to rotate within the skull. Although the brain is not rigidly attached to the skull interior, it can rotate around the relatively fixed brainstem. This rotation can lead to focal shear stresses and strains (31). Since the skull is most constraining around the foramen magnum and frontal compartments, particularly in the ventral region, this theory is supported by research revealing predominance of injuries to the anterior fossa, regardless of whether the original point of impact was to the frontal or occipital lobes (31).

As mentioned previously, the ability of the brain to move within the skull causes the brain and skull to move at different speeds upon impact or acceleration. When the brain lags behind the skull during linear acceleration, it “pushes” against the skull, causing an increase in the ICP at that location, and a decrease in ICP distal to it (28). The shearing from both the motion of the brain and these ICP pressure gradients can lead to axons stretching. The stretching causes enlargement of the axons at locations where microtubules are damaged, resulting in diffuse axonal injuries (DAI) (29).

An outstanding issue regarding blunt trauma and accelerative injuries is the relative importance of translational and rotational motion. Although initial research tended to focus primarily on translational motion, studies using both animal models and numerical modeling have shown that DAI, acute subdural hematoma and most other types of brain injury can be generated by pure rotational motion (32–34). Although rotational motion can cause these injuries in the absence of translational motion, most researchers agree that brain injuries are most often caused by a combination of shear stresses and strains associated with rotational motion *and* direct site contusions and elevated ICPs associated with translational motion (28, 35). Although, the first theory of rotational motion (differential motion of adjacent tissues) indicates that even low level rotations cause cortical damage, cortical lesions are typically attributed to translational motion, while brain stem injuries are attributed to shear strain (35).

Another factor in blunt trauma injuries is the direction of impact. First, localized injuries will be associated with the point of impact (coup) and the location distal to the point of impact (contrecoup). Thus, lateral impacts will tend to cause contusions in the temporal lobes of both hemispheres, and frontal and rear impacts will tend to cause contusions in both the frontal and occipital lobes. Second, the direction of impact will affect the relative motion of the two brain hemispheres. In a frontal impact, both hemispheres will move together as a single mass. Thus, higher angular accelerations are required to cause a TBI, and with bridging veins more vulnerable, there is a higher rate of acute subdural hematoma. In contrast, in a lateral impact the two brain hemispheres respond as two separate masses. Thus, with half the mass to move, lower levels of angular acceleration are required to cause a TBI, and DAI are more likely (36, 37).

### Blunt trauma thresholds

Existing head injury thresholds following blunt impact are based on results from animal studies and human cadaver studies. The simplest head injury threshold criterion is maximum translational acceleration. Maximum translational acceleration is obtained from drop tests in which the resultant acceleration is measured at the center of gravity of the head. The Federal Motor Vehicle Safety Standard (FMVSS) 571.218 (for motorcycle protection) uses a threshold of 400 g peak acceleration, which is interpreted as the limit above which there is significant risk of serious head and brain injury (38). The Snell Memorial Foundation sets forth a lower standard threshold for motorcycle helmets at 275 g peak acceleration (39). The US Army aircrew HGU-56/P helmet is designed to a standard of 175 g peak acceleration at the headband and 150 g peak acceleration at the crown (40). This criterion is based on US Army aviation accident data (40, 41). For these standards, the maximum translational acceleration criterion uses the International Standards Organization (ISO) head form (Figure 1C) and is designed to prevent skull fracture and subdural hematoma.

**Figure 2:**
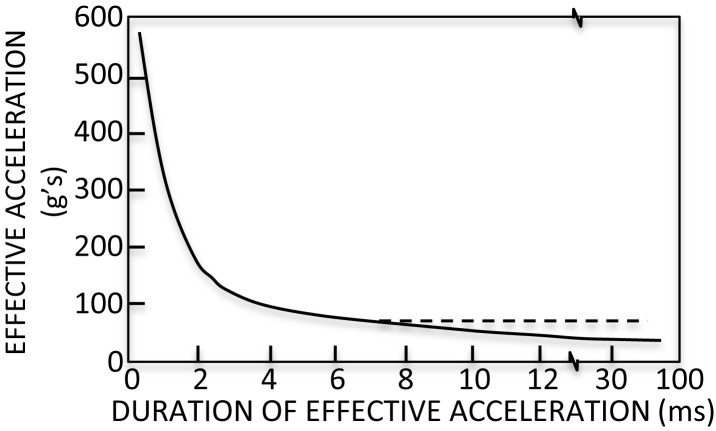
**Wayne State Tolerance Curve (WSTC)**. The WSTC predicts injury based upon the maximum acceleration over a given duration that can be tolerated without leading to a skull fracture. It was the first head injury criterion based upon biomechanical research (42).

To refine the peak acceleration injury criterion, the Wayne State University Cerebral Concussion Tolerance Curve (WSTC) was developed. The WSTC predicts injury as a function of the average acceleration and the duration of the acceleration. It is the first injury criterion to include findings from biomechanical research of head injury (42). The WSTC is an empirical relationship between the acceleration of the head and impact time (Figure 2). It describes the maximum acceleration over a given duration that can be tolerated without leading to a skull fracture. Data supporting the WSTC was derived from drop tests of embalmed cadaver heads, air-blasts to exposed cadaver brains, and hammer blows to animal heads (42).

The Head Injury Criterion (HIC) is the most commonly used injury criteria for designing protective measures to prevent skull fracture which, for this use, is considered a severe brain injury (43). The HIC is calculated as a function of the duration of acceleration at the center of gravity of the head (Eqn. 1), assuming that the head is a one-mass structure:

(1) $$\text{HIC}={\left\{\left(t_{\text{2}}-t_{\text{1}}\right){\left[\frac{1}{t_{\text{2}}-t_{\text{1}}}\int_{t_{1}}^{t_{\text{2}}}a\left(t\right)\text{dt}\right]}^{2.5}\right\}}_{\max}$$

where *t*_1_ is the initial time (s), *t*_2_ is the final time (*s*) and *a*(*t*) is the acceleration at the center of gravity of the head. The acceleration window (*t*_2-_*t*_1_) is usually 15 or 36 ms, depending upon the context of the impact. For most automotive applications, a 15 ms window is used, although a 36 ms window is more typically used in airbag testing. The HIC was proposed in 1970 based on the results of drop tests conducted in the late 1950s. The underlying data for the HIC is limited as it is based on 23 tests using five embalmed cadavers (44), and is calibrated to the Hybrid III head form (Figure 1D). In spite of the limited underlying dataset, the HIC has led to significant improvements in motor vehicle safety and protective head equipment design. It is, nevertheless, insufficient when applied to mild TBI with subtle underlying neuropathology. The National Highway Traffic and Safety Administration (NHTSA) standard is a HIC of 700 for ages six and up, and is based on a 15 ms window. The ISO also uses an acceleration window of 15 ms (45). The FMVSS-208 (for occupant crash protection) and FMVSS-213 (for child restraint systems) use a HIC of 700 as an injury threshold (46). In an effort to characterize the relationship between HIC values and mortality Prasad and Mertz (47) developed the Head Injury Risk Curve. This curve, based upon human cadaver test data, indicates that a HIC of 1400 gives a 50% probability of sustaining a life-threatening brain injury, and a HIC of 700 reduces the probability to 5% (48). Thus, the US federal HIC standards are well below the severe injury thresholds.

It should be noted that the WSTC, peak translational acceleration and the HIC are not the only injury criteria that have been developed for blunt trauma injuries. To name a few, the weighted principal component score (wPCS) has been proposed as a metric for evaluating the risk of mild TBI (49), the Gadd Severity Index remains a “current” standard for head injury protection, in spite being first proposed in 1966 (50), the head injury power (HIP) is based upon the generation rate of change of both the translational and rotational kinetic injury (51), and both the rotational injury criterion (RIC) and power rotational head injury criterion (PRHIC) are based upon angular acceleration (52). In spite of the plethora of alternative criteria available for blunt trauma, the HIC and peak translation acceleration criteria remain the most common metrics used for design and evaluation of protective gear, largely because standard test equipment and head surrogates have been calibrated to these criteria.

### Blunt trauma protection

Protecting the head against blunt impact trauma has been in practice throughout human history. For the past few decades the military and the sports communities have sought to identify padding or suspension systems that improve the blunt trauma protection provided by protective headgear. Regardless of the context, protecting the head against blunt impacts invariably has evolved to some combination of padding, load distribution, and standoff distance. For load distribution, most helmets start with an outer shell. For military helmets, the shell serves the dual purpose of both blunt impact and foreign body penetration protection. Below the outer shell is typically some type of energy absorbing foam. Since the rate and force of loading can differ greatly depending on the impact conditions, the density and thickness of the padding inside the shell also greatly differ. To protect the head from the great range of threats, helmet manufactures have turned to using a graded padding system comprised of a stack of pads of varying thicknesses and densities. The pads closer to the head typically have a lower density and are used to absorb the energy from low-velocity, low energy impacts. The pads at a greater distance from the head typically have a greater density and are capable of absorbing higher energy loads.

For contact sports, such as football, new helmet systems and rule changes have reduced the number of skull fractures, but it is unclear if the helmets have had any effect on the incidence of concussions (53). The helmet systems described above typically only reduce the linear acceleration associated with skull fractures and do not specifically address the effects of rotational acceleration. The current helmets also do not specifically address the effects of repeated impacts, where there is emerging evidence that repeated sub-injury threshold blows to the head can still cause mild to moderate TBI. The scientific understanding of the cause and effect relationship remains unclear.

## Blast

Blast injury is caused by exposure to a complex high air pressure environment (primary effects), ballistic impact from shrapnel and fragments (secondary effects), whole body acceleration, and subsequent impacts with objects such as walls or ground (tertiary effects). Exposure to primary blast effects (air overpressure) can, by itself, set up a series of concurrent events that can cause brain injury. These are the force of the shock wave impacting against the head, direct transmission of energy of the blast wave through the head and into the brain, and short duration accelerative motions of the head caused by temporal gradients in overpressure around the head. A detailed overview of air blast physics is beyond the scope of this paper. However, medical blast injury researchers will benefit from a review of topics, such as Mach stem formation, blast wave generation, and the relationship between static and dynamic overpressure. There are numerous sources that provide thorough descriptions, including *Blast Waves* (54).

Ultimately, injuries from exposure to blast, like blunt trauma and ballistic penetration, are fundamentally about transfer of energy from an external environment through the skull and into the brain. This energy transfer ultimately results in tissue damage through a number of proposed underlying mechanisms, including pressure loads to the torso leading to air emboli or upward fluid surge into the head (55–58), shear and stress waves inducing micro (cellular and subcellular) (59, 60) and macro (gross morphological) level damage (61), physical deformation or flexing of the skull caused by pressure gradients (62), cerebrospinal fluid micro-cavitation (63, 64), and acceleration of the brain against the inside of the skull (65, 66). At this time, none of the various theories of primary blast-induced TBI have been either conclusively discredited or substantially supported with empirical evidence. Thus, with a conspicuous absence of strong experimental data supporting any specific mechanism of cellular damage from blast exposure, one of the most fundamental issues surrounding blast-induced TBI is the remaining ambiguity about the mechanism of injury.

**Figure 3:**
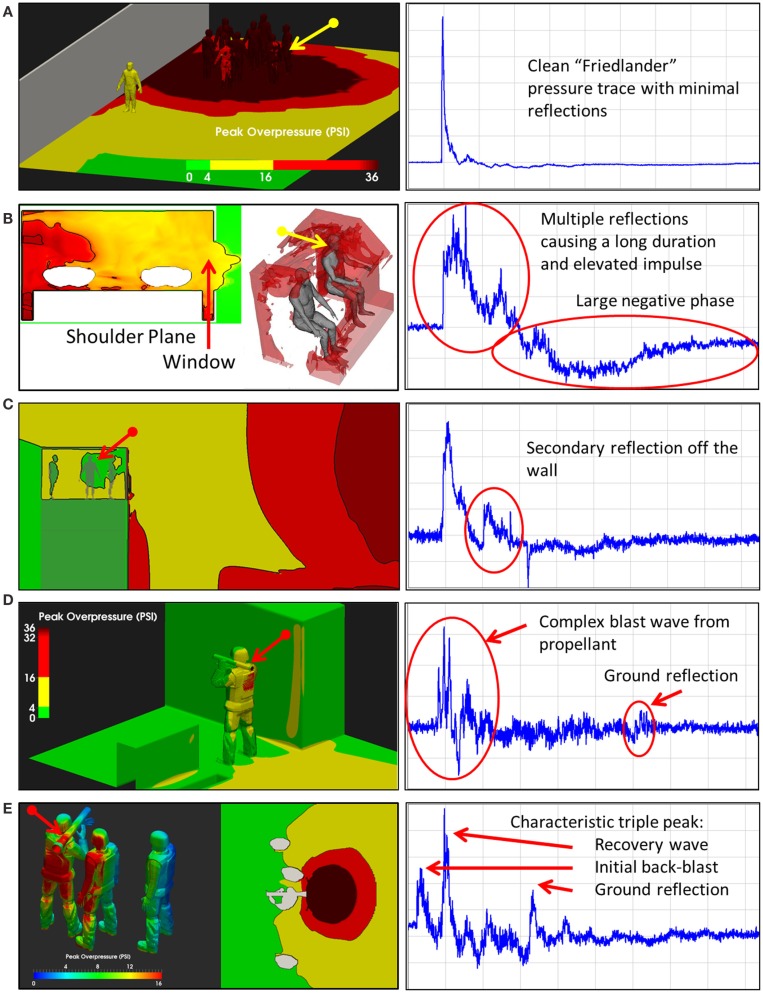
**Blast exposure scenarios**. As illustrated in these five scenes, the pressure-time history associated with an explosion varies considerably with respect to the environment in which the exposure occurs. Multiple reflective surfaces can increase the duration of exposure or cause multiple reflections, increasing the impulse imparted to an individual in the environment (Wiri S, Needham C Reconstruction of IED blast loading to personnel in the open. Twenty-first International Shock Interaction Symposium, International Shock Wave Institute, 3–8, August, 2014, Riga, Latvia). **(A)** Open field exposure measured with a shoulder mounted blast sensor. Bomb was about 3 feet above the ground. **(B)** Enclosed space, in the cab of a truck hit by an Rocket Propelled Grenade with the window open. Sensor on the back of the head. **(C)** Guard tower hit by Vehicle Bomb Improvised Explosive Device. Sensor mounted on the back of the head. **(D)** Shoulder fired rocked launcher (LAW). Sensor mounted on shoulder. **(E)** Shoulder fired Carl Gustav Recoiless Rifle. Sensor mounted on the back of the head.

When an individual is exposed to primary blast overpressure, there are infinite exposure scenarios. Exposure to an individual is a function not only of the overpressure, but also factors such as the presence of reflecting objects near the exposed individual (Figures 3B,C), the use of protective equipment such as helmets and face shields (67, 68), and unusual explosives (Figures 3D,E). Thus, given this enormous variation, to truly understand how blast waves may cause TBI, a fundamental understanding of how energy is deposited in the brain is necessary.

**Figure 4:**
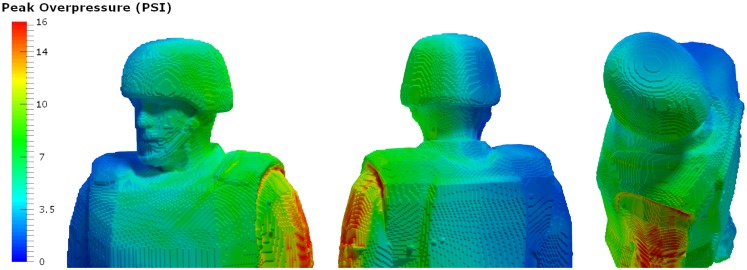
**Pressure loads simulated based on the exposure**. Computational analyses using the Second-Order Hydrodynamics with Automatic Mesh Refinement Code (SHAMRC) simulates explosive effects in complex environments, allowing prediction of static and dynamic overpressure as a function of time and location on the individual in a blast environment. In this case, blast loads on the left side of the head exceed the threshold for tympanic membrane rupture, while the loads on the rights side do not (Wiri S, Needham C Reconstruction of IED blast loading to personnel in the open. Twenty-first International Shock Interaction Symposium, International Shock Wave Institute, 3–8, August, 2014, Riga, Latvia).

A typical personnel-borne blast wave sensor overpressure trace usually contains multiple peaks, which are caused by reflections from the ground, structures, or nearby personnel. The explosive source and precise locations and orientation of the subject determine a unique overpressure waveform. Such a complex blast wave, interacting with the orientation and protective equipment of the exposed individual, will affect the amount of energy deposited into the head (69), as will the distribution of the energy as it hits the exposed individual. As seen in Figure 4, peak overpressures across the head and body can vary widely. The pressure variations determine how energy affects the body in terms of applied forces, which can induce high magnitude, short duration accelerations even without direct impact. In the scenario in Figure 3, the individual in the yellow zone (Figure 4) experienced extremely high overpressures on the shoulder and arm, which would probably have minimal physiological effect. However, exposures to the left ear exceeded 55 kPa, which is more than enough to potentially rupture the tympanic membrane (70). Although we often talk about a pressure differential across the head causing both head deformation (62) and acceleration (71), a more accurate representation of the physics is that an *impulse* (the integral of pressure over time) differential will cause both head deformation and acceleration. Counter-intuitively, for a given overpressure level a larger blast will have a longer positive phase duration so that the blast has time to wrap around the head, leading to a smaller impulse differential. Likewise, for a given blast, a shorter standoff distance will have both a higher overpressure and a shorter positive phase duration so that the blast does *not* have time to wrap around the head, leading to a larger impulse differential.

Once blast waves impact the head, computational modeling can provide significant insight into blast energy transmission through the skull and dissipation into brain tissue. Computational modeling indicates that the skull generally behaves as a low-pass filter. Live and cadaver animal tests have validated this finding, showing that the higher frequency content of the blast wave energy is removed as it enters the calvarium (72–74). Once the blast enters the skull, the effect on brain tissue is still under investigation. However, computer simulations and test data suggest that stress waves induce shear, spallation, implosion, and inertial effects within the brain tissue (69), leading to diffuse axonal injury in white matter regions (75). Models also suggest that the enhancement effects of pressure waves passing through the skull and blast reflection at the interface of different materials cause neuronal damage and tissue disruption, typically in the traditional coup and contrecoup regions (75).

An outstanding challenge to understanding the effects of blast on the brain is that stresses and strains within living brain tissue cannot be directly measured. The ICP has been identified as a useful surrogate metric for energy deposition. Based upon numerical modeling, peak stresses and strains in the brain correlated with the edges of high-pressure regions at the coup and contrecoup locations (65, 76). The ICP has the added benefit of correlation to clinical outcome (77).

One aspect of the blast environment that often goes unaddressed is the change in head inertia during the application of force from the blast wave during exposure. Even in porcine shock tube experiments in which deliberate effort is made to prevent acceleration of the head, accelerations in excess of 1,200 g have been measured over short durations under blast loads of 30 psi (~200 kPa) peak incident exposure (78). These accelerations are likely caused by extended dynamic pressures resulting from the experimental set up, but they were none-the-less a real outcome of these experimental blast exposures. Although these accelerations were measured over durations of 7 ms or less, the extreme magnitudes of acceleration imply a significant potential for injury. Previous studies have indicated that impact with the ground (secondary injury) would also result in higher global head accelerations, but this and other tests, supported by pathological data that lack reports of secondary insults, suggest otherwise. In field blast testing with Hybrid III test dummies, peak resultant accelerations of the center of gravity of the head have been as high as 500 g for 35–40 psi (~250–300 kPa) peak incident overpressure exposures with associated HICs well over the 50% injury level of 1,400 (79).

### Blast thresholds

Thresholds for injury are another major area of uncertainty with respect to blast-induced TBI. A number of test efforts have sought to identify criteria for outcomes ranging from apnea (80) to fatalities (81). Although there are a number of outstanding questions regarding the physics of the energy deposition in the brain, computational modeling that has been validated against test data has provided a first estimate for blast neurotrauma based upon the occurrence of apnea immediately following the blast (81).

In the absence of a validated blast neurotrauma criterion, many apply criteria such as the 150 g peak acceleration criterion or the HIC to estimate risk of head injury from accelerative loading. Unfortunately, both modeling and test data confirm that blast loads can induce very short acceleration durations (from <1 to 7 ms) with load levels in excess of 1000 g (79). These parameters are way beyond that for which any blunt trauma or acceleration criterion has been validated. Even aside from the issue of validated ranges, the mechanics of blast trauma are very different from that assumed in traditional blunt impact-based criteria, rendering the use of blunt trauma criteria in a blast environment inappropriate.

Clearly, the absence of a clear understanding of the mechanism of injury impedes development of validated blast neurotrauma injury criteria. Another major issue is the applicability of animal models. Is apnea, for example, a good outcome on which to base neurotrauma thresholds in humans? How does the onset of apnea correlate with severity of injury, probability of lethality, or probability of chronic deficits following the acute phase of injury? It is commonly understood that some form of scaling is required to equate injury thresholds from smaller animal data to humans. As a baseline, most researchers scale data based upon the relative mass of the animal to humans; however, scaling based upon skull thickness, brain mass, brain to body mass ratio or white to gray matter ratios may also be appropriate. Ultimately, a biophysical basis for scaling requires a rigorous understanding of both the physics of blast and the mechanism of injury.

### Blast protection

A number of studies have used both testing and simulation to assess the effects of protective equipment, primarily helmets and face shields in preventing blast-induced neurotrauma. Unfortunately, the TBI rates from recent conflicts have demonstrated that there remains a level of elevated risk (82). Studies have shown that injury thresholds for unprotected personnel are much higher for neurotrauma than they are for barotrauma to lungs and other hollow viscera. However, when the torso is protected, the threshold for barotrauma injury is raised, resulting in fewer barotrauma deaths and a higher prevalence of blast-induced TBI (82). Thus, torso protection has significantly increased tolerance to exposure, but the unintended consequence of reduced pulmonary vulnerability appears to be greater risk of TBI.

A recent study looking into the theory of blast exposure inducing micro-cavitation in the cerebrospinal fluid provided clear evidence of the protective effects of helmets. Under direct blast exposure, a helmet significantly reduced the measured ICP at both the frontal (coup) and occipital (contrecoup) regions of a human cadaver head (63). Although the mechanism by which this protective effect is obtained is unclear from these tests, it can be concluded that providing coverage of the head reduces the amount of energy transferred to brain. Further studies have used computational models to demonstrate the additional protective effects of face shields and novel padding materials in providing additional reductions in the ICP inside the brain (22, 67, 83).

Ultimately, the goal is to provide optimized protective equipment that reduces the risk and severity of blast-induced TBI in known, quantitative ways. Unfortunately, current test methods are insufficient, which makes attaining this goal a major challenge. Computational modeling will be necessary to complete this task, but the lack of validity in brain injury estimates based on external loading conditions require the continued use of live animal models to explore neurotrauma questions. Numerous animal models have been used over the years, but are limited in their ability to assess the effect of a helmet on preserving neurological function. Several recent porcine studies have demonstrated this challenge, as pig skulls are significantly thicker than humans, and ICPs measured in the calvarium of a pig under blast loads show considerable attenuation not seen with similar human head tests (74, 76, 78), even when the pig head was exposed to the side to reduce the effective skull thickness facing the blast.

Although specific injury criteria relating to this mechanism are lacking, significant protection can be attained by blocking the blast wave from reaching the skull. Simulations have shown that current helmets can reduce transmitted energy, but not completely (68). Due to the need to hear, see and breathe, current helmet configurations do not fully shield the head. Future developers will need to find the appropriate compromise between head coverage, mobility, weight, and visibility for the wearer to optimize this protection.

## Discussion

Although significant efforts have been made studying TBI, a clinically effective neurospecific therapy remains elusive. It is widely understood that the mechanisms underlying this condition need to be elucidated. To that end, the biochemical and neuropathological characterization of this condition is being advanced. Though incomplete, there has been remarkable progress. One important mechanistic aspect of TBI that has not received as much attention and, thus, effort is in understanding the physics of the inciting insult. This is critical if clinically meaningful mitigation or treatment strategies are to be developed.

Historically, since live testing at thresholds causing injury is unethical, research approaches to studying TBI have been limited to cadaver and animal testing. Cadaver testing provides valuable information regarding the response of the bone structure to insult and can be informative in understanding how loads change as they pass through the skull and into the brain cavity. However, changes in the tissue properties of the brain happen rapidly upon death so that it is difficult to get meaningful data about transmission of loads through the brain tissue using cadavers. The lack of a physiological response also substantially limits how much information about neurotrauma can be gleaned from cadaver tests. Animal testing has the advantage of providing a means of obtaining physiological response data. However, anatomical and brain function differences limit the validity of these data, and proven methods of scaling from animals to humans are lacking. Given limitations of existing test methods, injury criteria are generally based on indirect measures and global rigid-body kinematics. As a result, there is limited understanding of the specific mechanical trauma to brain tissues that lead to TBI.

Although additional research is needed to better characterize the effects of missile fragmentation, modern ballistic protection for the head is adequate, as it is based on the rule that any head penetration is unacceptable. However, behind armor blunt trauma remains an issue, particularly as we search for lighter and more versatile protection. There is no scientific basis for linking clay indentation to brain injury, as is the approach used in current testing of military ballistic helmets (25, 26). The use of clay for measuring behind armor blunt trauma originated with abdominal impacts and was validated with animal data, and the human skull is not capable of the same degree of transient deformation as the abdomen. No comparable tests have been performed to validate clay deformation for behind armor head trauma (26).

Current blunt trauma injury criteria have been invaluable to reducing the incidence of skull fractures and hematomas. However, for the range of other types of brain injuries that occur from blunt impact or acceleration, injury criteria are still critically lacking. Computer simulations, head surrogates and real-time acceleration data collection have been used in an attempt to correlate external accelerations with internal brain stresses (65, 84–86). Ongoing research with collegiate football teams (49) and joint Department of Defense and National Football League data collection efforts should result in prospective data that will provide valuable insights into the threshold loads for brain injuries that occur in the absence of a skull fracture. A major challenge facing these and other blunt trauma injury studies, however, is ambiguity in the definition and diagnosis of *mild* TBI, which makes it difficult to correlate measured accelerations or forces with consistent levels of injury. This challenge is further exacerbated in human studies by the fact that the susceptibility of the brain changes with repeated impacts (87), and research participants in the sports and military populations are often *not* naïve to concussion. Thus, variability in concussion histories becomes an important and, often, poorly specified confounding factor in determining potential induced vulnerability from using data from these populations.

The primary challenge to preclinical TBI research is uncertainty regarding both injury models. While this challenge impacts ballistic and blunt trauma research, it is most acutely an issue with blast neurotrauma. Across industry, academia and government research institutions, there are a plethora of injury models, creating inconsistencies in load levels, methods for applying loads and outcome measures. As a consequence, the results of these studies are confusing and, often, conflicting. As an example, a significant issue in blast neurotrauma research is replication of blast loading environments in a laboratory setting. Although free-field blast testing is, of course, most realistic, many institutions lack access to blast test sites, and the free-field blast environment presents particular challenges with respect to both instrumentation and animal handling. Shock or blast tubes are the most common approach to simulating the blast environment in a laboratory setting. Although shock tubes are relatively inexpensive and have proliferated in the last decade of blast neurotrauma research, many tubes are used incorrectly, with tremendous consequences to our understanding of blast effects on the brain. Placement of test subjects in front of the tube, rather than inside it, is the most common error seen in blast tube research. Because the shock wave rapidly expands outside the tube and is followed by a jetting of cold air, a test subject placed at this location is exposed to significant dynamic loading. The large dynamic loads induce head accelerations that are erroneously attributed to blast overpressure. Although the significance of this error is sometimes dismissed, recent porcine blast experiments in which the specimen was placed in front of a tube resulted in head accelerations in excess of 1,200 g. Not surprisingly, those subjects with peak accelerations over 1000 g had a 50% risk of apnea (5/10), indicative of possible neurotrauma. No subjects with less than 1000 g peak acceleration experienced apnea (78). In contrast, in an ongoing test program led by one of the authors, porcine subjects placed fully inside the shock tube resulted in negligible head accelerations and little evidence of neurological trauma, even with more than double the peak overpressure of the other tests. Until the community adopts consistent, physics-based test standards, major outstanding issues will remain, such as the fundamental question of whether the overpressure contribution to blast-induced TBI is even a significant factor relative to the effects of acceleration.

As eluded to earlier, another issue with current blast neurotrauma test methods is scaling outcomes from animal models to humans. In blast, the air blast phenomena scale uses the cube root of the energy for free field parameters. Thus, the duration of the positive phase of the blast loading must be adjusted for the size and type of the test subject. A simple heuristic for scaling is that the positive phase duration should be between the time it takes to engulf the target and ten times that duration. Body mass scaling has been the standard for several decades, having been validated in the 1960s in a large test program studying the relationship between blast-induced pulmonary injuries and survivability (88). Unfortunately, while body mass scaling is certainly necessary in neurotrauma research, it is unclear whether it is sufficient. Scaling by head mass, brain mass, skull thickness or other physical properties may be necessary to equate animal test data to humans (89). Aside from simple positive phase duration heuristic, proper scaling requires an understanding of the underlying injury mechanism. In the absence of a clear understanding of the injury mechanism, appropriate scaling laws cannot be established. And, in the absence of appropriate scaling, there can be no confidence in estimating human injury thresholds using data collected from animal models.

## Conclusion

Although TBI has been researched for over 40 years, meaningful brain specific therapy has not been realized. Thus, it remains an important field of research for the development of both treatment and prevention. It is important for civilian, law enforcement and military communities. In many preclinical studies, attention to the physics of the injurious event is not adequately considered. This must first be addressed when developing a surrogate model of the human condition and also in fully understanding that condition.

For ballistic protection, physiologically-based behind helmet blunt trauma criteria are needed. The most pressing knowledge gap in blunt trauma is criteria for mild and moderate TBI – i.e., injuries that do *not* involve skull fractures or subdural hematomas. There is also a tremendous need for a quantitative relationship between injury thresholds and the number and frequency of repeated impacts. A better understanding of the mechanisms of injury is needed for both blast and blunt trauma insults, although this knowledge gap is most significant for blast neurotrauma. Clarity on the mechanisms of injury is prerequisite to development of valid injury criteria and scaling laws for animal models.

To an unfortunate extent, research into blunt, blast and ballistic neurotrauma has been disjointed, with engineers and medical professionals pursuing the issues of prevention, mitigation, diagnosis and therapeutics with far less multidisciplinary collaboration than is desirable. Particularly as we begin to appreciate the heterogeneity and clinical complexities of mild TBI, a multidisciplinary approach to TBI research will be essential to addressing both the engineering issues of prevention and mitigation and the medical issues of diagnosis and therapeutics. Although the existing knowledge gaps are tremendous, advancements in neurotrauma research will profoundly impact the quality of life for both TBI victims and their families.

## Conflict of Interest Statement

The opinions belonging to and expressed by Geoffrey Ling herein belong solely to him. They do not and should not be interpreted as being those of or endorsed by any agency of the US government. The authors declare that the research was conducted in the absence of any commercial or financial relationships that could be construed as a potential conflict of interest.

## References

[1] Faul M Xu L Wald M Coronodo V Traumatic Brain Injury in the United States: Emergency Department Visits, Hospitalizations and Deaths 2002-2006. Atlanta, GA: Denters for Disease Control and Prevention, National center for Injury Prevention and Control (2010).

[2] Theeler BJ Flynn FG Erickson JC. Headaches after concussion in US soldiers returning from Iraq or Afghanistan. Headache (2010) 50(8):1262–72. 10.1111/j.1526-4610.2010.01700.x 20553333

[3] Kraus JF McArthur DL Epidemiology of brain injury. 2 ed In: Evans RW, editor. Neurology and Trauma. New York, NY: Oxford University Press (2006). 201 p.

[4] Fackler ML Gunshot wound review. Ann Emerg Med (1996) 28:194–203 10.1016/S0196-0644(96)70062-8 8759585

[5] DiMaio VJ Copeland AR Besant-Matthews PE Fletcher LA Jones A Minimal velocities necessary for perforation of skin by air gun pellets and bullets. J Forensic Sci (1982) 27(4):894–8. 7175470

[6] Reilly P Bullock R Head Injury 2Ed: Pathophysiology & Management. 2nd ed Boca Raton, FL: Taylor & Francis (2005).

[7] Law S. Thickness and resistivity variations over the upper surface of the human skull. Brain Topogr (1993) 6(2):99–109. 10.1007/BF01191074 8123431

[8] Ruan J Prasad P. The effects of skull thickness variations on human head dynamic impact responses. Stapp Car Crash J (2001) 45: 395–414. 17458755 10.4271/2001-22-0018

[9] Maas AIR Dearden M Teasdale GM Braakman R Cohadon F Iannotti F EBIC-guidelines for management of severe head injury in adults. Acta Neurochir (1997) 139(4):286–94. 10.1007/BF01808823 9202767

[10] Levin HS Neurobehavioral Consequences of Closed Head Injury. Oxford University Press (1982).

[11] Moreira-Gonzalez A Papay FE Zins JE. Calvarial thickness and its relation to cranial bone harvest. Plast Reconstr Surg (2006) 117(6):1964–71. 10.1097/01.prs.0000209933.78532.a7 16651971

[12] Yoganandan N Frontiers in Head and Neck Trauma: Clinical and Biomechanical. Amsterdam: IOS Press (1998).

[13] Sturdivan L Cutting Mechanisms. Handbook of Human Vulnerability. Edgewood Arsenal, MD: Department of Army Headquarters (1976).

[14] Ryan JM Cooper GJ Maynard RL. Wound ballistics: contemporary and future research. J R Army Med Corps (1988) 134(3):119–25. 10.1136/jramc-134-03-02 3057188

[15] Fackler ML. Ballistic injury. Ann Emerg Med (1986) 15(12):1451–5. 10.1016/S0196-0644(86)80941-6 3777618

[16] Fackler ML. Wound ballistics: a review of common misconceptions. JAMA (1988) 259(18):2730–6. 10.1001/jama.1988.03720180056033 3282087

[17] Hollerman JJ Fackler ML Coldwell DM Ben-Menachem Y. Gunshot wounds: 1. Bullets, ballistics, and mechanisms of injury. Am J Roentgenol (1990) 155(4):685–90. 10.2214/ajr.155.4.2119095 2119095

[18] Kazim SF Shamim MS Tahir MZ Enam SA Waheed S. Management of penetrating brain injury. J Emerg Trauma Shock (2011) 4(3):395–402. 10.4103/0974-2700.83871 21887033 PMC3162712

[19] Lenhart MK Savitsky E Eastbridge B Combat Casualty Care: Lessons Learned from OEF and OIF. Fort Detrick, MD: Government Printing Office (2012).

[20] Yoganandan N Pintar FA Zhang J Gennarelli T Beuse N Biomechanical aspects of blunt and penentrating head injuries. In: Gilchrist MD, editor. IUTAM Symposium on Impact Biomechanics: From Fundamental Insights to Applications. Solid Mechanics and Its Applications. 124 Netherlands: Springer (2005). p. 173–84.

[21] MacPherson D Bullet Penetration: Modeling the Dynamics and the Incapacitation Resulting from Wound Trauma. Ballistic Publications (1994).

[22] Kulkarni SG Gao XL Horner SE Zheng JQ David NV Ballistic helmets – their design, materials, and performance against traumatic brain injury. Compos Struct (2013) 101:313–31 10.1016/j.compstruct.2013.02.014

[23] Freitas CJ Mathis JT Scott N Bigger RP MacKiewicz J. Dynamic response due to behind helmet blunt trauma measured with a human head surrogate. Int J Med Sci (2014) 11(5):409–25. 10.7150/ijms.8079 24688303 PMC3970092

[24] Vargas-Gonzalez L Walsh SM Wolbert J Impact and Ballistic Response of Hybridized Thermoplastic Laminates. Aberdeen Proving Ground, MD: Army Research Laboratory (2011). DTIC ADA538498.

[25] Standard, N. I. J. “0106.01 for Ballistic Helmets.” December 1981 (1981).

[26] Review of Department of Defense Test Protocols for Combat Helmets. Test protocols for backface deformation: statistical considerations and assessment. Committee on Review of Test Protocols Used by the DoD to Test Combat Helmets Board on Army Sciences and Technology DoEaPS, National Research Council. Washington, DC: National Academies Press (2014). 25077182

[27] Bass C Walilko T Kent R Evaluation of Head Surrogates for the Assessment of Explosive and Ballistic Injuries. CTTSO Technical Support Working Group (2011). Available from: http://www.tswg.gov

[28] Post A Hoshizaki TB Mechanisms of brain impact injuries and their prediction: a review. Trauma (2012) 14(4):327–49 10.1177/1460408612446573

[29] King AI. Fundamentals of impact biomechanics: part I – biomechanics of the head, neck, and thorax. Annu Rev Biomed Eng (2000) 2(1):55. 10.1146/annurev.bioeng.2.1.55 11701507

[30] Ommaya AK Gennarelli TA Cerebral concussion and traumatic unconsciousness. Correlation of experimental and clinical observations of blunt head injuries. Brain (1974) 97(4):633–54 10.1093/brain/97.1.633 4215541

[31] Hardy WN Khalil TB King AI Literature review of head injury biomechanics. Int J Impact Eng (1994) 15(4):561–86 10.1016/0734-743X(94)80034-7

[32] Adams JH Graham DI Gennarelli TA Acceleration induced head injury in the monkey. II. neuropathology. In: Jellinger K Gullotta F Mossakowski M, editors. Experimental and Clinical Neuropathology. Acta Neuropathologica Supplementum. 7. Berlin: Springer (1981). p. 26–8. 10.1007/978-3-642-81553-9_8 6939248

[33] Adams JH Graham DI Gennarelli TA Head injury in man and experimental animals: neuropathology. In: Adams JH, editor. Trauma and Regeneration. Acta Neurochirurgica Supplementum. 32. Vienna: Springer (1983). p. 15–30. 10.1007/978-3-7091-4147-2_2 6581702

[34] Gennarelli TA Abel JM Adams H Graham D Differential Tolerance of Frontal and Temporal Lobes to Contusion Induced by Angular Acceleration. SAE Technical Paper No. 791022 1979 p.

[35] Zhou C Khalil TB King AI A New Model Comparing Impact Responses of the Homogeneous and Inhomogeneous Human Brain. SAE Technical Paper (1995).

[36] Kleiven S. Influence of impact direction on the human head in prediction of subdural hematoma. J Neurotrauma (2003) 20(4):365–79. 10.1089/089771503765172327 12866816

[37] Ommaya AK Thibault L Bandak FA Mechanisms of impact head injury. Int J Impact Eng (1994) 15(4):535–60 10.1016/0734-743X(94)80033-6

[38] Transportation UDo. Federal Motor Vehicle Safety Standard 2018. CFR 49. Washington, DC (1988). 2 p.

[39] Snell. 2015 Helmet Standard for Protective Headgear. For Use in Motorcycles and Other Motorized Vehicles. North Highlands, CA: Snell Memorial Foundation (2015).

[40] McEntire BJ Phillip W Blunt Impact Performance Characteristics of the Advanced Combat Helmet and the Paratrooper and Infantry Personnel Armor System for Ground Troops Helmet, No. USAARL-2005-12. Fort Rucker, AL: US Army Aeromedical Research Laboratory (2005).

[41] Slobodnik BA. SPH-4 helmet damage and head injury correlation. Aviat Space Environ Med (1979) 50(2):139–46. 444173

[42] Hutchinson J Kaiser MJ Lankarani HM The head injury criterion (HIC) functional. Appl Math Comput (1998) 96(1):1–16 10.1016/S0096-3003(97)10106-0

[43] Versace J A Review of the Severity Index. No. 710881. SAE Technical Paper (1971).

[44] Lissner HR Lebow M Evans FG Experimental studies on the relation between acceleration and intracranial pressure changes in man. Surg Gynecol Obstet (1960) 111:329–38. 14417481

[45] Eppinger R Sun E Bandak F Haffner M Khaewpong N Maltese M Development of Improved Injury Criteria for the Assessment of Advanced Automotive Restraint Systems–II. Washington, DC: National Highway Traffic Safety Administration (1999). p. 1–70.

[46] Administration NHTS Occupant Crash Protection. Title 49 Code of Federal Regulations (CFR) Part 571, Section 208. Washington, DC: Office of the Federal Register, National Archives and Records Administraiton (2000).

[47] Prasad P Mertz HJ The position of the United States delegation to the ISO Working Group 6 on the use of HIC in the automotive environment. No. 851246. SAE Technical Paper (1985).

[48] Mertz HJ Prasad P Irwin AL Injury Risk Curves for Children and Adults in Frontal and Rear Collisions. No. 973318. SAE Technical Paper (1997).

[49] Greenwald RM Gwin JT Chu JJ Crisco JJ. Head impact severity measures for evaluating mild traumatic brain injury risk exposure. Neurosurgery (2008) 62(4):789. 10.1227/01.neu.0000318162.67472.ad 18496184 PMC2790598

[50] Gadd CW Use of a Weighted-Impulse Criterion for Estimating Injury Hazard. No. 660793 SAE Technical Paper (1966).

[51] Newman JA Shewchenko N Welbourne E. A proposed new biomechanical head injury assessment function-the maximum power index. Stapp Car Crash J (2000) 44:215–47. 17458729 10.4271/2000-01-SC16

[52] Kimpara H Iwamoto M. Mild traumatic brain injury predictors based on angular accelerations during impacts. Ann Biomed Eng (2012) 40(1):114–26. 10.1007/s10439-011-0414-2 21994065

[53] Daneshvar DH Nowinski CJ McKee AC Cantu RC The epidemiology of sport-related concussion. Clin Sports Med (2011) 30(1):1–17 10.1016/j.csm.2010.08.006 21074078 PMC2987636

[54] Needham CE Blast Waves Shock Wave and High Pressure Phenomena. Berlin: Springer (2010).

[55] Cernak I Savic J Malicevic Z Zunic G Radosevic P Ivanovic I Involvement of the central nervous system in the general response to pulmonary blast injury. J Trauma Surgery (1996) 40(3S):100S–4S. 10.1097/00005373-199603001-00023 8606388

[56] Lemonick DM Bombings and blast injuries: a primer for physicians. Am J Clin Med (2011) 8(3):134–40.

[57] Courtney AC Courtney MW. A thoracic mechanism of mild traumatic brain injury due to blast pressure waves. Med Hypotheses (2009) 72(1):76–83. 10.1016/j.mehy.2008.08.015 18829180

[58] Chen Y Huang W. Non-impact, blast-induced mild TBI and PTSD: concepts and caveats. Brain Inj (2011) 25(7–8):641–50. 10.3109/02699052.2011.580313 21604927

[59] Taylor PA Ford CC. Simulation of blast-induced early-time intracranial wave physics leading to traumatic brain injury. J Biomech Eng (2009) 131(6):061007. 10.1115/1.3118765 19449961

[60] Kucherov Y Hubler GK DePalma RG. Blast induced mild traumatic brain injury/concussion: a physical analysis. J Appl Phys (2012) 112(10):104701. 10.1089/neu.2011.1895 22026588

[61] Chafi MS Karami G Ziejewski M. Biomechanical assessment of brain dynamic responses due to blast pressure waves. Ann Biomed Eng (2010) 38(2):490–504. 10.1007/s10439-009-9813-z 19806456

[62] Moss WC King MJ Blackman EG. Skull flexure from blast waves: a mechanism for brain injury with implications for helmet design. Phys Rev Lett (2009) 103(10):108702. 10.1103/PhysRevLett.103.108702 19792349

[63] Goeller J Wardlaw A Treichler D O’Bruba J Weiss G. Investigation of cavitation as a possible damage mechanism in blast-induced traumatic brain injury. J Neurotrauma (2012) 29(10):1970–81. 10.1089/neu.2011.2224 22489674

[64] Taylor PA Ludwigsen JS Ford CC Investigation of blast-induced traumatic brain injury. Brain Inj (2014) 28(7):879–95 10.3109/02699052.2014.888478 24766453 PMC4046872

[65] Zhang L Yang KH King AI. A proposed injury threshold for mild traumatic brain injury. J Biomech Eng (2004) 126(2):226–36. 10.1115/1.1691446 15179853

[66] Risling M Plantman S Angeria M Rostami E Bellander BM Kirkegaard M Mechanisms of blast induced brain injuries, experimental studies in rats. Neuroimage (2011) 54(Suppl 1):S89–97. 10.1016/j.neuroimage.2010.05.031 20493951

[67] Nyein M Jerusalem A Radovitzky R Moore D Noels L Modeling Blast-Related Brain Injury. DTIC Document. Cambridge: Massachusetts Institute of Technology (2008).

[68] Nyein MK Jason AM Yu L Pita CM Joannopoulos JD Moore DF In silico investigation of intracranial blast mitigation with relevance to military traumatic brain injury. Proc Natl Acad Sci USA (2010) 107(48):20703–8. 10.1073/pnas.1014786107 21098257 PMC2996433

[69] Elder GA Mitsis EM Ahlers ST Cristian A Blast-induced mild traumatic brain injury. Psychiatr Clin North Am (2010) 33(4):757–81 10.1016/j.psc.2010.08.001 21093677

[70] Baker WE Kulesz JJ Westine PS Cox PA Wilbeck JS A manual for the prediction of blast and fragment loadings on structures. No. SWRI-02-5594. San Antonio TX: Southwest Research Institute (1981)

[71] Tan XG Przekwas AJ Rule G Iyer K Ott K Merkle A, editors. Modeling articulated human body dynamics under a representative blast loading. ASME 2011 International Mechanical Engineering Congress and Exposition Denver, CO (2011).

[72] Clemedson C-J Pettersson H Propagation of a high explosive air shock wave through different parts of an animal body. Am J Physiol (1955) 184(1):119–26. 13283101 10.1152/ajplegacy.1955.184.1.119

[73] Chavko M Koller WA Prusaczyk WK McCarron RM. Measurement of blast wave by a miniature fiber optic pressure transducer in the rat brain. J Neurosci Methods (2007) 159(2):277–81. 10.1016/j.jneumeth.2006.07.018 16949675

[74] Säljö A Arrhén F Bolouri H Mayorga M Hamberger A. Neuropathology and pressure in the pig brain resulting from low-impulse noise exposure. J Neurotrauma (2008) 25(12):1397–406. 10.1089/neu.2008.0602 19146459

[75] Dagro AM McKee PJ Kraft RH Zhang TG Satapathy SS A Preliminary Investigation of Traumatically Induced Axonal Injury in a Three-Dimensional (3-D) Finite Element Model (FEM) of the Human Head During Blast-Loading. DTIC Document. No. ARL-TR-6504. Aberdeen Proving Ground, MD: US Army Research Laboratory (2013).

[76] Zhu F Skelton P Chou CC Mao H Yang KH King AI. Biomechanical responses of a pig head under blast loading: a computational simulation. Int J Numer Method Biomed Eng (2013) 29(3):392–407. 10.1002/cnm.2518 23345257

[77] Sundstrom T Grande P Juul N Kock-Jensen C Romner B Wester K, editors. Management of Severe Traumatic Brain Injury: Evidence, Tricks and Pitfalls. New York, NY: Springer Science & Business Media (2012).

[78] Shridharani JK Wood GW Panzer MB Capehart BP Nyein MK Radovitzky RA Porcine head response to blast. Front Neurol (2012) 3:70 10.3389/fneur.2012.00070 22586417 PMC3347090

[79] Lockhart P Cronin D Williams K Ouellet S. Investigation of head response to blast loading. J Trauma (2011) 70(2):E29–36. 10.1097/TA.0b013e3181de3f4b 20664376

[80] Rafaels KA Cameron R Panzer MB Salzar RS Woods WA Feldman SH Brain injury risk from primary blast. J Trauma (2012) 73(4):895–901 10.1097/TA.0b013e31825a760e 22836001

[81] Rafaels K Bass CR Salzar RS Panzer MB Woods W Feldman S Survival risk assessment for primary blast exposures to the head. J Neurotrauma (2011) 28(11):2319–28. 10.1089/neu.2009.1207 21463161

[82] Bass CR Panzer MB Rafaels KA Wood G Shridharani J Capehart B Brain injuries from blast. Ann Biomed Eng (2012) 40(1):185–202 10.1007/s10439-011-0424-0 22012085

[83] Sharma S Makwana R Zhang L Evaluation of blast mitigation capability of advanced combat helmet by finite element modeling. 12th International LS-DYNA Users Conference Detroit (2012).

[84] King AI Yang KH Zhang L Hardy W Viano DC, editors. Is head Injury Caused by Linear or Angular Acceleration. In: The IRCOBI conference 2003 Lisbon (2003). p. 1–12.

[85] Duma SM Manoogian SJ Bussone WR Brolinson PG Goforth MW Donnenwerth JJ Analysis of real-time head accelerations in collegiate football players. Clin J Sport Med (2005) 15(1):3–8. 10.1097/00042752-200501000-00002 15654184

[86] Pellman EJ Viano DC Tucker AM Casson IR. Concussion in professional football: location and direction of helmet impacts – Part 2. Neurosurgery (2003) 53(6):1328–41. 10.1227/01.NEU.0000093499.20604.21 14633299

[87] Guskiewicz KM McCrea M Marshall SW Cantu RC Randolph C Barr W Cumulative effects associated with recurrent concussion in collegiate football players: the NCAA Concussion Study. JAMA (2003) 290(19):2549–55. 10.1001/jama.290.19.2549 14625331

[88] Bowen IG Fletcher ER Richmond DR Estimate of Man’s Tolerance to the Direct Effects of Air Blast. Albuquerque, NM: Lovelace Foundation for Medical Education and Research (1968).

[89] Panzer MB Wood GW Bass CR. Scaling in neurotrauma: how do we apply animal experiments to people? Exp Neurol (2014) 261(0):120–6. 10.1016/j.expneurol.2014.07.002 25035134

